# To: Catecholamines and serum potassium alterations in critically ill neonates: a prospective cohort study

**DOI:** 10.62675/2965-2774.20260180

**Published:** 2026-08-21

**Authors:** Viji Gunasekaran, Thirunavukkarasu Arun Babu

**Affiliations:** 1 All India Institute of Medical Sciences Department of Pediatrics Andhra Pradesh India Department of Pediatrics, All India Institute of Medical Sciences - Mangalagiri, Andhra Pradesh, India.

**TO THE EDITOR**

The study by Silva et al. explores the association between catecholamine administration and serum potassium alterations in critically ill neonates, a clinically relevant but underexplored topic in neonatal intensive care.^(1)^ Both hypokalemia and hyperkalemia are common in neonatal intensive care units and may result in serious complications including arrhythmias, neurological injury, and increased mortality. Although the study attempts to address an important knowledge gap, several limitations reduce the strength and generalizability of its conclusions.

A major strength is the prospective cohort design, which permits temporal assessment between drug exposure and electrolyte disturbances. Daily collection of medication and laboratory data improves consistency, and the use of mixed-effects regression analysis to account for repeated potassium measurements is statistically appropriate. However, the study remains exploratory because several important confounding variables were inadequately controlled.

One major limitation is the lack of detailed information regarding catecholamine dosage, infusion duration, timing of potassium measurements, and severity of illness. Catecholamines, particularly dopamine, exhibit dose-dependent receptor activity and physiological effects.^(2)^ Without dose stratification, it is difficult to determine whether potassium disturbances were caused by the drugs themselves or by the underlying severity of shock requiring vasopressor therapy. Neonates receiving norepinephrine are often more critically ill, with metabolic acidosis, tissue hypoperfusion, renal dysfunction, or multiorgan failure, all of which independently contribute to hyperkalemia. Thus, confounding by indication remains a significant concern.

The study also inadequately evaluated other important determinants of potassium homeostasis such as acid-base imbalance, acute kidney injury, hemolysis, blood transfusions, parenteral nutrition, potassium supplementation, and adrenal dysfunction. Although estimated glomerular filtration rate and urine output were included in the regression model, these markers alone are insufficient to comprehensively assess renal function in critically ill neonates.

External validity is another limitation because the study was conducted in a single tertiary center in Brazil, where clinical practices and vasoactive drug protocols may differ from other institutions. In addition, the norepinephrine subgroup was relatively small, with only 17 neonates receiving the drug. Therefore, conclusions regarding its hyperkalemic potential should be interpreted cautiously.

The observational design also prevents causal inference. The findings demonstrate association rather than definitive causation. Furthermore, clinically important outcomes such as arrhythmias, dyskalemia related mortality, or need for potassium correction therapy were not assessed, limiting the clinical applicability of the results. The relatively low reported incidence of hyperkalemia compared with previous neonatal literature also raises concerns regarding patient selection, laboratory timing, or local management protocols.

Despite these limitations, the study provides preliminary evidence that catecholamines may influence neonatal potassium balance and emphasizes the importance of close electrolyte monitoring during vasoactive therapy. Overall, the findings should be interpreted cautiously and considered hypothesis generating rather than definitive evidence of catecholamine induced potassium disturbances in critically ill neonates.
